# Associations between smoking and alcohol consumption with blood pressure in a middle-aged population

**DOI:** 10.18332/tid/162440

**Published:** 2023-05-18

**Authors:** Alexandre Vallée

**Affiliations:** 1 Department of Epidemiology-Data-Biostatistics, Delegation of Clinical Research and Innovation (DRCI), Foch Hospital, France

**Keywords:** alcohol, tobacco, blood pressure, hypertension, cardiovascular disease

## Abstract

**INTRODUCTION:**

Inconsistent association between tobacco smoking, alcohol consumption and hypertension have been highlighted. The purpose of our study was to investigate the associations between smoking use and alcohol with systolic and diastolic blood pressure (SBP, DBP) and hypertension in a middle-aged population.

**METHODS:**

Smoking status was based on smoking pack-years and cigarettes per day, and alcohol consumption was measured in units/day. Gender associations between smoking and alcohol consumption with BP and hypertension were estimated using multiple linear regressions. Synergistic effects between smoking and alcohol were investigating in both genders.

**RESULTS:**

A total of 290913 individuals of the UK Biobank population were included (133950 men and 156963 women). Current smoking was significantly associated with lower SBP, DBP and lower hypertension prevalence, in both genders (p<0.001). However, cigarettes per day were associated with higher SBP in men current smokers [B=0.05 (0.02), p<0.001] with higher hypertension (p=0.001) but not with DBP (p=0.205). Similar results were observed in women current smokers [SBP: B=0.10 (0.02), p<0.001; DBP, p=0.217 and hypertension, p=0.019]. The number of smoking pack-years was only associated with higher levels in SBP in men (p=0.047) and in women (p<0.001). In both genders, alcohol consumption was associated with higher SBP, DBP and hypertension (p<0.001). Synergistic effects were observed for alcohol consumption on smoking pack-years and cigarettes per day with SBP and DBP.

**CONCLUSIONS:**

Smoking and alcohol were associated with higher BP in current smokers with synergistic effects. The findings suggest the importance of considering smoking and alcohol consumption in BP control in addition to antihypertensive medication and public health practice.

## INTRODUCTION

Hypertension (HTN) was the main risk factor for global burden and accounted for more than 9 million deaths in 2010^1^. In parallel, tobacco smoking is one of the major public health challenge and was responsible for more than 6 million deaths per year worldwide^1^. The relation between blood pressure (BP) and tobacco habits remained unclear among studies showing a positive^2^ or a negative association^3^. The association between tobacco smoking and low BP could be explained by different behaviors or socioeconomic factors^4^. Current smokers may have a lower body mass index (BMI) than non-smokers, which could explain this negative association^5^. Nevertheless, other epidemiological studies showed that smoking tobacco was associated with high BP^6^. The combination of tobacco smoking and high BP may have a synergistic effect on cardiovascular events^7^. However, few studies have focused on smoking impact on BP levels in current smokers.

In parallel, recent guidelines recommended to limit daily alcohol consumption to two or fewer drinks per day for men and one drink for women^8^. A positive association between heavy drinking and hypertension has been found^9^; however, this relationship remains unclear, especially in women.

Many people both smoke and drink, and many chemical pathways should reinforce this association^10^. The combination of smoking and alcohol intake is associated with increased risk of mortality^11^. Patterns of both drinking and smoking are highly socially associated, even if health public policies fight these unhealthy behaviors. Few studies have focused on these combinations on hypertension and BP in the general population. Thus, the purpose of this study was to investigate the associations between tobacco smoking and alcohol consumption with BP and hypertension, and their combinations, in a middle-aged population.

## METHODS

### UK Biobank population

The UK Biobank is a prospective cohort for the investigation, prevention, diagnosis, and treatment of chronic diseases, such as cardiovascular (CV) diseases in adults. A total of 502478 Britons from the UK National Health Service Register were included between 2006 and 2010, across 22 UK cities. The cohort was phenotyped and genotyped from participants who responded to a questionnaire and had a computer-assisted interview, from their physical and functional measures, and who provided blood, urine, and saliva samples. Data included socioeconomic, behavior and lifestyle, mental health battery, clinical diagnoses and therapies, genetics, imaging, and physiological biomarkers from blood and urine samples. The cohort protocol can be found in the literature^12^.

### Study population

**Figure 1:**
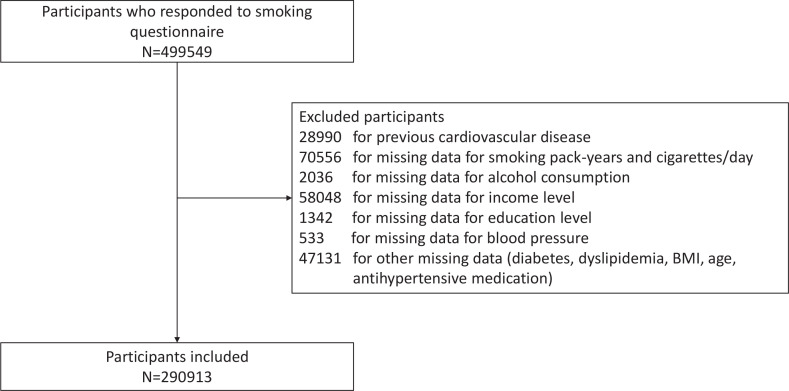
Flowchart of participant selection

In all, 499549 volunteers of the UK Biobank who responded to the questionnaire on smoking status were recruited. We excluded 28990 participants with previous CV events from the analyses due to the interaction between tobacco smoking and CV disorders; 179646 participants were excluded for missing data, and we thus analyzed 290913 individuals in this study (Figure 1).

### Blood pressure measurement

Systolic (SBP) and diastolic blood pressure (DBP) were measured twice at the assessment center by the use of an automated BP device (Omron 705 IT electronic blood pressure monitor; OMRON Healthcare Europe B.V. Kruisweg 577 2132 NA Hoofddorp) or manually by the use of a sphygmomanometer with an inflatable cuff in association with a stethoscope if the blood pressure device failed to measure the BP or if the largest inflatable cuff of the device did not fit around the individual’s arm.

The participant sat in a chair for all the measures. The measures were carried out by nurses trained in measuring BP. Available multiple measurements for one participant were averaged. The Omron 705 IT BP monitor satisfied the Association for the Advancement of Medical Instrumentation SP10 standard and was validated by the British Hypertension Society protocol, with an overall ‘A’ grade for both SBP and DBP^13^. Nevertheless, automated devices measure higher BP in comparison to manual sphygmomanometers, thus, we adjusted both SBP and DBP that were measured using the automated device using the algorithms^14^:

*SBP=3.3171+0.92019×SBP (mmHg)+6.02468×sex coefficient*

and

*DBP=14.5647+0.80929×DBP (mmHg)+2.01089×sex coefficient*

where the *sex coefficient* =1 for males and 0 for females.

### Covariates

Diabetes status was defined on either receiving anti-diabetic medication or diabetes diagnosed by a doctor or a fasting glucose concentration ≥7mmol/L. Dyslipidemia was defined as having a fasting plasma total-cholesterol or triglycerides level of ≥6.61 mmol/L (255 mg/dL) or >1.7 mmol/L (150 mg/dL), respectively, or having statins medication. Medications were characterized by the question: ‘Do you regularly take any of the following medications?’.

Hypertension was defined as SBP of at least 140 mmHg and/or DBP of at least 90 mmHg, according to guidelines by the European Society of Cardiology, and/or antihypertensive drug used, or hypertension diagnosed by a doctor. CV diseases were defined by heart attack, angina, and stroke, as diagnosed by a doctor, and reported in questionnaires. Body mass index (BMI) was calculated as weight (kg) divided by height-squared (m^2^) and categorized as: high >30, moderate 25–30, and low <25 kg/m^2^). Biological parameters were detailed in the UK Biobank protocol. Education level was defined in three categories: high (college or university degree); intermediate (A/AS levels or equivalent, O levels/GCSEs or equivalent, other profession qualification, e.g. nursing, teaching etc.); and low (none of the aforementioned). Yearly income level (in £) was defined as: high, >52000; moderate, 18000–51999; and low, <18000.

### Smoking status

Participants were categorized by self-report, as ‘current’, ‘past’ or ‘never’ smokers. Current tobacco smokers were defined as participants who responded ‘yes, on most or all days’ or ‘yes, only occasionally’ to the question: ‘Do you smoke tobacco now?’. Smoking pack-years were calculated for individuals who have ever smoked. Smoking pack-years were calculated as the average number of packs smoked per day multiplied by the total number of years of smoking in lifetime. The general definition of a pack-year is the number of cigarettes smoked per day, divided by twenty, multiplied by the number of years of smoking. In the UK Biobank, the number of years of smoking is calculated by subtracting the age of starting smoking from the age smoking was stopped (or age at inclusion for current smokers), using the equation:

*Pack-years = Number of cigarettes per day/20×(age stopped smoking - age started smoking)*

For current smokers, the participants had to respond to: ‘About how many cigarettes do you smoke on average per day?’; and for past smokers: ‘About how many cigarettes did you smoke on average per day?’. Participants who responded ‘never smoked’ were allocated zero for both smoking pack-years and cigarettes per day.

### Alcohol consumption

Although the alcohol questionnaire has not been formally validated, several studies have shown expected associations with alcohol^15^. For alcohol drinker status, participants had to responded for their alcohol status: ‘current’, ‘past’, or ‘never’. Then, participants self-reported the number of alcohol units (10 mL of pure ethanol) consumed, in ‘units per week’ or ‘units per month’ (for less frequent drinkers), across numerous beverage categories (red wine, white wine/champagne, beer/cider, spirits, fortified wine, or other). The UK Biobank assessment defined units of alcohol as: a pint or can of beer/lager/cider=two units; a 25 mL single shot of spirits=one unit; and a standard glass of wine (175 mL) =two units. The number of weekly units was computed by summing all the units consumed in all categories in a week. When reported monthly, the intake was converted to units per week by dividing by 4.3. The number of weekly units was divided by 7 to determine units per day. Participants who responded ‘past’ or ‘never’ were allocated zero for daily alcohol consumption according to the UK Biobank.

### Statistical analysis

Characteristics of the study population were described as mean with standard deviation (SD) for continuous variables. Categorical variables were described as number and percentage. Statistical analyses were stratified by gender since hypertension differs between men and women^16^ and a difference in tobacco consumption between gender was observed^17^. Comparisons between all groups of smoking status were performed using ANOVA tests.

Association between smoking status, smoking pack-years or cigarettes per day with alcohol status or alcohol consumption per day, and blood pressure levels (SBP and DBP), were examined with linear regression models, computing regression coefficients (B) with standard error (SE), adjusted for Model 1: antihypertensive medication + age; Model 2: model 1 + BMI; and Model 3: model 2 + diabetes, dyslipidemia, education level, and income level.

Associations between smoking status and alcohol consumption with hypertension prevalence were examined with logistic regression models with odds ratio (OR) and 95% confidence interval (CI), adjusted for Model 1: antihypertensive medication + age; Model 2: model 1 + BMI; and Model 3: model 2 + diabetes, dyslipidemia, education level and income level. Interactions were examined by including simultaneous alcohol consumption per day and smoking pack-years or cigarettes per day and their interaction term. Relationships between smoking and alcohol consumption with SBP, DBP, and hypertension were investigated in each subgroup, i.e. current, past, or never smokers. To investigate the synergistic effects between smoking pack-years/cigarettes per day and alcohol consumption on blood pressure (SBP and DBP) in current smokers, the differences in correlation were assessed using Steiger’s Z test between the adjusted individuals and combined models. Statistics were performed using SAS software (version 9.4; SAS Institute, Carry, NC). A p<0.05 was considered statistically significant.

## RESULTS

A total of 290913 individuals were included for analysis, with 133950 men and 156963 women. When stratified by smoking status, there were among men: 14350 (10.9%) current smokers, 38674 (29.9%) past smokers, and 80746 (60.3%) never smokers; and among women: 13505 (8.6%) current smokers, 35169 (22.4%) past smokers, and 108289 (61.0%) never smokers (Table 1). For both genders, current smokers were younger (p<0.001), had lower BMI (p<0.001), lower hypertension prevalence (p<0.001), and lower SBP and DBP (p<0.001). However, while men showed higher levels of alcohol consumption in current smokers (p<0.001), this was not the case for women (i.e. among past smokers, p<0.001) (Table 1).

**Table 1:** Characteristics* of the study population (N=290913)

*Characteristics*	*Men (N=133950)*							*Women (N=156963)*						
	*Current smokers (N=14530)*		*Past smokers (N=38674)*		*Never smokers (N=80746)*		*p*	*Current smokers (N=13505)*		*Past smokers (N=35169)*		*Never smokers (N=108289)*		*p*
**Age** (years)	54.2	8.2	58.3	7.6	55.0	8.1	<0.001	53.8	7.8	57.0	7.5	55.3	8.0	<0.001
**BMI** (kg/m^2^)	27.0	4.3	28.5	4.1	27.4	4.1	<0.001	26.6	5.1	27.6	5.24	26.8	5.1	<0.001
**BMI level**							<0.001							<0.001
High	3042	20.94	11764	30.42	17493	21.66		2931	21.70	9352	26.59	24007	22.17	
Moderate	6639	45.69	19662	50.84	40052	49.60		4788	35.45	13592	38.65	38744	35.78	
Low	4849	33.37	7248	18.74	23201	28.73		5786	42.84	12225	34.76	45538	42.05	
**SBP** (mmHg)	137	16	141	16	138	16	<0.001	124	17	128	17	127	18	<0.001
**DBP** (mmHg)	84	8	85	8	85	8	<0.001	79	8	80	8	80	8	<0.001
**Antihypertensive medication**	2272	15.64	10376	26.83	14408	17.84	<0.001	1786	13.22	6223	17.69	16607	15.34	<0.001
**Hypertension**	7488	51.53	25023	64.70	43115	53.40	<0.001	4367	32.34	14009	39.83	39700	36.66	<0.001
**Income**							<0.001							<0.001
High	2748	18.91	9742	25.19	27355	33.88		1858	13.76	7501	21.33	27996	25.85	
Moderate	7245	49.86	20796	53.77	41157	50.97		6603	48.89	18459	52.49	56972	52.61	
Low	4537	31.23	8136	21.04	12234	15.15		5044	37.35	9209	26.18	23321	21.54	
**Education level**							<0.001							<0.001
High	3403	23.42	11489	29.71	34308	42.49		2997	22.19	10845	30.84	40160	37.09	
Moderate	6781	46.67	17878	46.23	33343	41.29		6512	48.22	16633	47.29	50010	46.18	
Low	4346	29.91	9307	24.07	13095	16.22		3996	29.59	7691	21.87	18119	16.73	
**Diabetes**	1096	7.54	4165	10.77	5239	6.49	<0.001	718	5.32	2063	5.87	5417	5.00	<0.001
**Dyslipidemia**	9482	65.26	26582	68.73	47723	59.10	<0.001	7550	55.91	18635	52.99	50662	46.78	<0.001
**Alcohol status**							<0.001							<0.001
Current	13590	93.53	36828	95.23	75683	93.73		12289	91.00	32965	93.73	97840	90.35	
Past	727	5.00	1587	4.10	2037	2.52		778	5.76	1579	4.49	3046	2.81	
Never	213	1.47	259	0.67	3026	3.75		438	3.24	625	1.78	7403	6.84	
**Smoking pack-years**	28.7	19.6	23.0	19.4	-	-	<0.001	24.2	15.3	17.6	14.2	-	-	<0.001
**Cigarettes per day**	16.7	9.0	20.9	11.2	-	-		13.8	7.3	16.7	8.3	-	-	<0.001
**Alcohol consumption per day**	3.8	4.1	3.5	3.2	2.4	2.5	<0.001	1.9	2.7	2.1	2.2	1.2	1.6	<0.001

* Categorical variables in number and percentage, continuous variables in mean and standard deviation. BMI: body mass index. SBP: systolic blood pressure. DBP: diastolic blood pressure.

In the men population, smoking pack-years was negatively and significantly associated with SBP [Model 3: B= -0.02 (0.01), p<0.001], with DBP [Model 3: B= -0.04 (0.01), p<0.001] and with hypertension (Model 3: OR=0.98; 95% CI: 0.97–0.99, p<0.001) (Table 2). Similar results were observed between cigarettes per day and SBP (Model 3: B= -0.02 (0.01), p<0.001), DBP (Model 3: B= -0.03 (0.01), p<0.001), but not with hypertension (p=0.895). Compared to never smokers, current smokers showed negative and significant association with SBP [Model 3: B= -0.51 (0.10), p<0.001], with DBP [Model 3: B= -0.32 (0.05), p<0.001] and with hypertension (OR=0.91; 95% CI: 0.86–0.94, p<0.001). Current drinking was significantly associated with higher SBP [Model 3: B=2.00 (0.12), p<0.001], DBP [Model 3: B=0.92 (0.06), p<0.001], and hypertension (Model 3: OR=1.26; 95% CI: 1.17–1.36, p<0.001). Same results were observed with alcohol consumption per day with SBP (p<0.001), DBP (p<0.001) and with hypertension (p<0.001) (Table 2).

**Table 2:** Multiple linear and logistic regression modelsa of systolic, diastolic blood pressure and hypertension among men (N=133950)

*Variables*	*Model for tobacco status (Ref. Never smokers)*								*Model for alcohol status (Ref. Never drinkers)*					
	*Smoking pack-years*		*Cigarettes/day*		*Past smokers*		*Current smokers*		*Alcohol consumption/day*		*Past drinkers*		*Current drinkers*	
	*B (SE)*	*p*	*B (SE)*	*p*	*B (SE)*	*p*	*B (SE)*	*p*	*B (SE)*	*p*	*B (SE)*	*p*	*B (SE)*	*p*
**SBP**														
Unadjusted model^b^	0.06 (0.01)	<0.001	0.09 (0.01)	<0.001	1.97 (0.07)	<0.001	-1.26 (0.09)	<0.001	0.65 (0.01)	<0.001	-1.19 (0.18)	<0.001	2.22 (0.12)	<0.001
Model 1	0.03 (0.01)	<0.001	0.04 (0.01)	<0.001	0.93 (0.07)	<0.001	-0.56 (0.10)	<0.001	0.64 (0.01)	<0.001	-1.28 (0.17)	<0.001	2.04 (0.11)	<0.001
Model 2	-0.01 (0.002)	<0.001	-0.03 (0.01)	<0.001	0.49 (0.07)	<0.001	-0.24 (0.09)	0.006	0.55 (0.07)	<0.001	-1.31 (0.17)	<0.001	1.98 (0.12)	<0.001
Model 3	-0.02 (0.01)	<0.001	-0.02 (0.01)	<0.001	0.51 (0.07)	<0.001	-0.51 (0.10)	<0.001	0.63 (0.02)	<0.001	-1.42 (0.17)	<0.001	2.00 (0.12)	<0.001
**DBP**														
Unadjusted model^b^	0.01 (0.001)	0.154	0.01 (0.01)	0.172	0.45 (0.04)	<0.001	-1.19 (0.18)	<0.001	2.22 (0.12)	<0.001	-1.19 (0.18)	<0.001	2.22 (0.12)	<0.001
Model 1	0.01 (0.001)	0.122	0.01 (0.01)	<0.001	0.46 (0.04)	<0.001	-0.50 (0.05)	<0.001	0.33 (0.01)	<0.001	-1.28 (0.18)	<0.001	2.04 (0.12)	<0.001
Model 2	-0.01 (0.001)	<0.001	-0.01 (0.01)	<0.001	0.10 (0.04)	0.005	-0.25 (0.05)	<0.001	0.31 (0.01)	<0.001	-1.31 (0.17)	<0.001	1.98 (0.12)	<0.001
Model 3	-0.04 (0.01)	<0.001	-0.03 (0.01)	<0.001	0.11 (0.04)	0.002	-0.32 (0.05)	<0.001	0.32 (0.01)	<0.001	-0.70 (0.09)	<0.001	0.92 (0.06)	<0.001
**Hypertension**	**OR (95% CI)**	**p**	**OR (95% CI)**	**p**	**OR (95% CI)**	**p**	**OR (95% CI)**	**p**	**OR (95% CI)**	**p**	**OR (95% CI)**	**p**	**OR (95% CI)**	**p**
Unadjusted model^b^	1.01 (1.00–1.02)	<0.001	1.02 (1.01–1.03)	<0.001	1.60 (1.56–1.64)	<0.001	0.93 (0.90–0.96)	<0.001	1.07 (1.06–1.08)	<0.001	1.16 (1.06–1.27)	0.001	1.22 (1.14–1.31)	<0.001
Model 1	1.01 (1.00–1.02)	<0.001	1.01 (1.00–1.01)	<0.001	1.31 (0.28–1.35)	<0.001	0.98 (0.94–1.02)	0.251	1.08 (1.07–1.09)	<0.001	1.11 (1.01–1.21)	0.034	1.17 (1.09–1.26)	<0.001
Model 2	1.01 (1.00–1.02)	<0.001	1.01 (1.00–1.02)	<0.001	1.16 (1.13–1.19)	<0.001	1.02 (0.98–1.06)	0.401	1.07 (1.06–1.08)	<0.001	1.08 (0.98–1.19)	0.127	1.17 (1.09–1.26)	<0.001
Model 3	0.98 (0.97–0.99)	<0.001	1.00 (0.99–1.01)	0.895	1.09 (1.07–1.13)	<0.001	0.91 (0.86–0.94)	<0.001	1.08 (1.07–1.09)	<0.001	1.06 (0.96–1.16)	0.276	1.26 (1.17–1.36)	<0.001

a Associations were adjusted for Model 1: antihypertensive medication + age; Model 2: model 1 + BMI; and Model 3: model 2 + diabetes, dyslipidemia, educational and income levels.

b Model only adjusted for antihypertensive medication.

In the women population, smoking pack-years was negatively and significantly associated with SBP [Model 3: B= -0.07 (0.01), p<0.001], with DBP [Model 3: B=-0.03 (0.01), p<0.001], and with hypertension (Model 3: OR=0.99; 95% CI: 0.98–0.99, p<0.001) (Table 3). Similar results were observed between cigarettes per day and SBP [Model 3: B= -0.10 (0.01), p<0.001], with DBP [Model 3: B=-0.04 (0.01), p<0.001], and with hypertension (Model 3: OR=0.99; 95% CI: 0.98–0.99, p<0.001). Compared to never smokers, current smokers showed negative and significant association with SBP [Model 3: B= -1.31 (0.10), p<0.001], with DBP [Model 3: B= -0.38 (0.05), p<0.001], and with hypertension (OR=0.83; 95% CI: 0.79–0.86, p<0.001). Current drinking was significantly associated with higher SBP [Model 3: B=1.04 (0.09), p<0.001], with DBP [Model 3: B=0.57 (0.05), p<0.001], but not with hypertension (p=0.682). Same results were observed with alcohol consumption per day with SBP (p<0.001) and DBP (p<0.001), but with significant association with hypertension (p<0.001) (Table 3).

**Table 3:** Multiple linear and logistic regression models of systolic, diastolic blood pressure and hypertension among women (N=156963)

	*Model for tobacco status (Ref. Never smokers)*								*Model for alcohol status (Ref. Never drinkers)*					
*Variables*	*Smoking pack-years*		*Cigarettes/day*		*Past smokers*		*Current smokers*		*Alcohol consumption/day*		*Past drinkers*		*Current drinkers*	
	*B (SE)*	*p*	*B (SE)*	*p*	*B (SE)*	*p*	*B (SE)*	*p*	*B (SE)*	*p*	*B (SE)*	*p*	*B (SE)*	*p*
**SBP**														
Unadjusted model^b^	0.02 (0.01)	<0.001	0.01 (0.01)	0.611	1.37 (0.08)	<0.001	-2.20 (0.10)	<0.001	0.38 (0.02)	<0.001	-1.46 (0.17)	<0.001	0.33 (0.10)	0.001
Model 1	-0.02 (0.01)	<0.001	-0.03 (0.01)	<0.001	0.23 (0.08)	0.002	-1.13 (0.10)	<0.001	0.50 (0.02)	<0.001	-1.43 (0.16)	<0.001	0.71 (0.10)	<0.001
Model 2	-0.04 (0.01)	<0.001	-0.05 (0.01)	<0.001	-0.01 (0.07)	0.989	-1.01 (0.10)	<0.001	0.58 (0.06)	<0.001	-1.54 (0.15)	<0.001	0.95 (0.10)	<0.001
Model 3	-0.07 (0.01)	<0.001	-0.10 (0.01)	<0.001	0.09 (0.07)	0.203	-1.31 (0.10)	<0.001	0.69 (0.02)	<0.001	-1.36 (0.15)	<0.001	1.04 (0.09)	<0.001
**DBP**														
Unadjusted model^b^	0.001(0.001)	0.068	-0.01 (0.01)	0.368	0.19 (0.04)	<0.001	-0.49 (0.04)	<0.001	0.29 (0.01)	<0.001	-1.46 (0.17)	<0.001	0.33 (0.10)	0.001
Model 1	-0.01 (0.001)	<0.001	-0.01 (0.01)	0.058	0.12 (0.05)	0.001	-0.42 (0.04)	<0.001	0.30 (0.01)	<0.001	-1.44 (0.16)	<0.001	0.71 (0.10)	<0.001
Model 2	-0.02 (0.01)	<0.001	-0.02 (0.01)	<0.001	-0.08 (0.04)	0.031	-0.32 (0.05)	<0.001	0.38 (0.01)	<0.001	-1.54 (0.15)	<0.001	0.95 (0.10)	<0.001
Model 3	-0.03 (0.01)	<0.001	-0.04 (0.01)	<0.001	-0.06 (0.04)	0.079	-0.38 (0.05)	<0.001	0.41 (0.01)	<0.001	-0.55 (0.07)	<0.001	0.57 (0.05)	<0.001
**Hypertension**	**OR (95% CI)**	**p**	**OR (95% CI)**	**p**	**OR (95% CI)**	**p**	**OR (95% CI)**	**p**	**OR (95% CI)**	**p**	**OR (95% CI)**	**p**	**OR (95% CI)**	**p**
Unadjusted model^b^	1.01 (1.00–1.02)	<0.001	1.01 (1.00–1.02)	<0.001	1.14 (1.12–1.17)	<0.001	0.83 (0.79–0.86)	<0.001	1.01 (0.99–1.02)	0.658	0.88 (0.82–0.94)	<0.001	0.76 (0.73–0.79)	<0.001
Model 1	1.01 (1.00–1.01)	<0.001	1.01 (1.00–1.01)	<0.001	1.00 (0.98–1.03)	0.732	0.92 (0.88–0.96)	<0.001	1.02 (1.01–1.03)	<0.001	0.92 (0.85–0.99)	0.018	0.84 (0.80–0.88)	<0.001
Model 2	0.99 (0.98–1.00)	0.235	0.99 (0.98–0.99)	0.038	0.94 (0.91–0.96)	<0.001	0.92 (0.89–0.96)	<0.001	1.04 (1.03–1.05)	<0.001	0.92 (0.85–0.99)	0.023	0.91 (0.87–0.96)	<0.001
Model 3	0.99 (0.98–0.99)	<0.001	0.99 (0.98–0.99)	<0.001	0.91 (0.89–0.94)	<0.001	0.83 (0.79–0.86)	<0.001	1.06 (1.05–1.07)	<0.001	0.95 (0.88–1.03)	0.209	1.01 (0.96–1.06)	0.682

a Associations were adjusted for Model 1: antihypertensive medication + age; Model 2: model 1 + BMI; and Model 3: model 2 + diabetes, dyslipidemia, educational and income levels.

b Model only adjusted for antihypertensive medication.

Significant interactions were observed between smoking status and alcohol status in both genders (men, p<0.001; and women, p=0.075), smoking pack-years and alcohol consumption per day (men, p=0.022; and women, p<0.001), and between cigarettes per day and alcohol consumption per day (men, p=0.007; and women, p<0.001).

**Figure 2:**
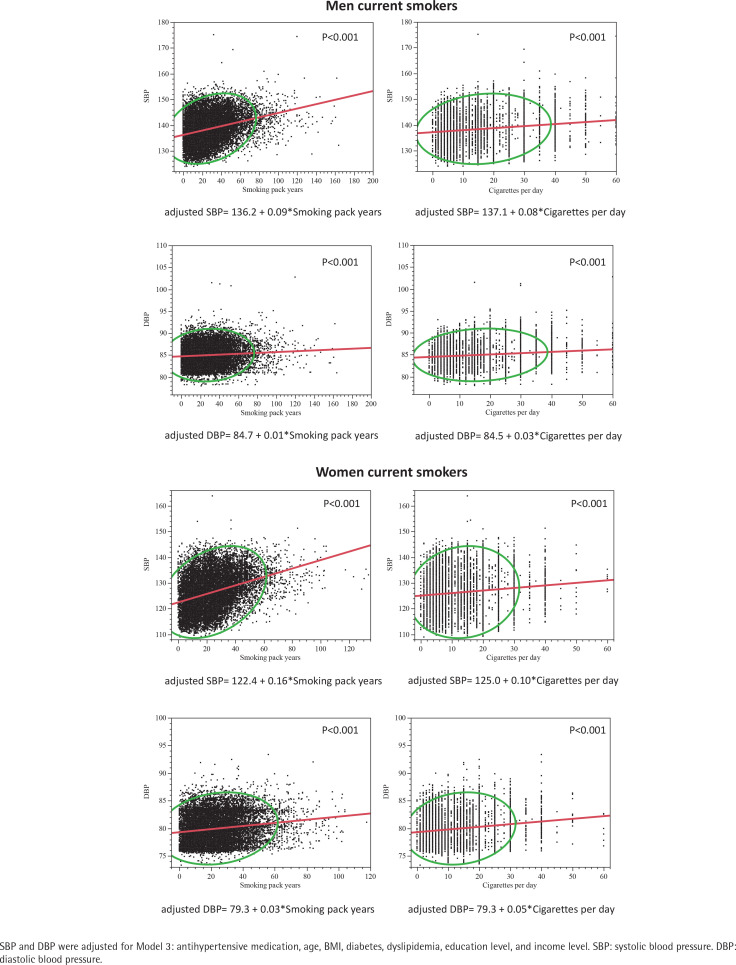
Linear regressions between SBP and DBP with smoking pack-years and cigarettes per day in men and women current smokers

**Figure 3:**
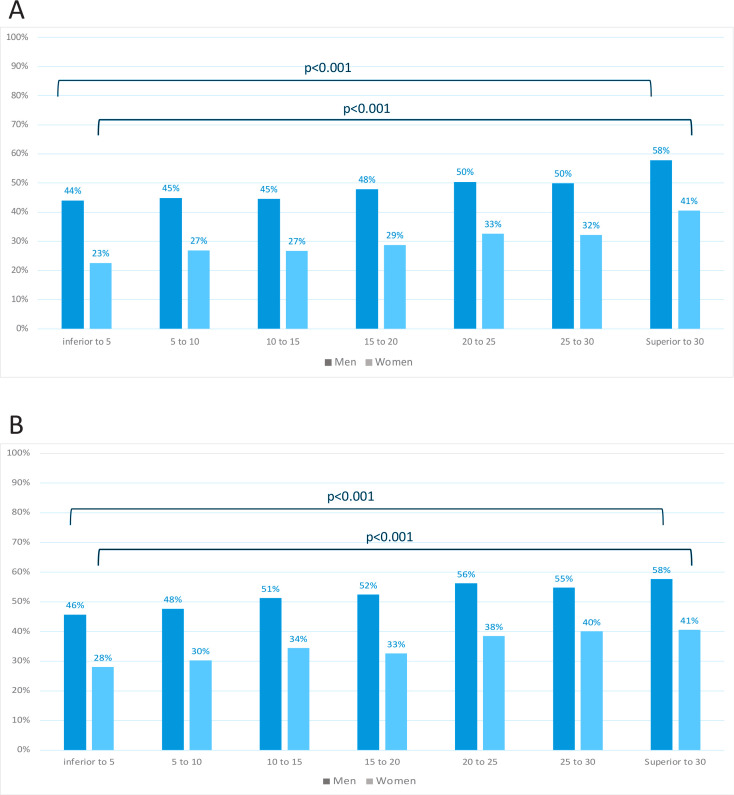
Hypertension prevalence among men and women current smokers according to: A) subgroups of number smoking pack-years, and B) subgroups of number cigarettes per day

Among men current smokers, a significant association was observed between SBP and smoking pack-years [Model 3: B=0.01 (0.001), p=0.047] but not with DBP (p=0.054) and hypertension prevalence (p=0.248) (Table 4). Similar results were observed between cigarettes per day with SBP (p=0.001) and DBP (p=0.205), but showing a significant association with hypertension (Model 3: OR=1.01; 95% CI: 1.00–1.02, p<0.001). SBP and DBP, adjusted for Model 3, showed linear significant correlations with both smoking pack-years (p<0.001) and cigarettes per day (p<0.001) among current men and women smokers (Figure 2). Men current smokers with >30 smoking pack-years showed higher proportion of hypertension (Figure 3). Similar results were observed between adjusted SBP and DBP with cigarettes per day (Figure 3).

**Table 4:** Associations* between SBP, DBP and hypertension and smoking pack-years, cigarettes/day and alcohol consumption/day in each group of current, past and never smokers for men (N=133950) and for women (N=156963)

*Tobacco status*	*Parameters (Model 3)*	*Systolic blood pressure*		*Diastolic blood pressure*		*Hypertension*	
		*B (SE)*	*p*	*B (SE)*	*p*	*OR (95% CI)*	*p*
**Men**							
Current	Smoking pack-years	0.01 (0.001)	0.047	0.01 (0.01)	0.054	1.00 (0.99–1.01)	0.248
	Cigarettes/day	0.05 (0.02)	0.001	0.01 (0.01)	0.205	1.01 (1.00–1.02)	0.001
	Alcohol consumption/day	0.66 (0.03)	<0.001	0.32 (0.02)	<0.001	1.09 (1.08–1.10)	<0.001
Past	Smoking pack-years	-0.01 (0.01)	0.097	-0.01 (0.002)	<0.001	1.01 (0.99–1.02)	0.059
	Cigarettes/day	0.02 (0.01)	0.006	0.01 (0.01)	0.113	1.01 (1.00–1.02)	<0.001
	Alcohol consumption/day	0.69 (0.02)	<0.001	0.31 (0.01)	<0.001	1.09 (1.08–1.10)	<0.001
Never	Smoking pack-years	NA	NA	NA	NA	NA	NA
	Cigarettes/day	NA	NA	NA	NA	NA	NA
	Alcohol consumption/day	0.58 (0.02)	<0.001	0.32 (0.01)	<0.001	1.07 (1.06–1.08)	<0.001
**Women**							
Current	Smoking pack-years	0.04 (0.01)	<0.001	0.01 (0.01)	0.188	1.01 (1.00–1.01)	0.176
	Cigarettes/day	0.10 (0.02)	<0.001	0.01 (0.01)	0.217	1.01 (1.00–1.02)	0.019
	Alcohol consumption/day	0.81 (0.05)	<0.001	0.42 (0.02)	<0.001	1.09 (1.07–1.10)	<0.001
Past	Smoking pack-years	-0.02 (0.01)	0.122	-0.02 (0.01)	<0.001	1.00 (0.99–1.01)	0.178
	Cigarettes/day	-0.01 (0.01)	0.264	0.01 (0.01)	0.714	1.00 (0.99–1.01)	0.058
	Alcohol consumption/day	0.84 (0.04)	<0.001	0.44 (0.02)	<0.001	1.07 (1.06–1.08)	<0.001
Never	Smoking pack-years	NA	NA	NA	NA	NA	NA
	Cigarettes/day	NA	NA	NA	NA	NA	NA
	Alcohol consumption/day	0.61 (0.03)	<0.001	0.41 (0.01)	<0.001	1.05 (1.04–1.06)	<0.001

* Associations were adjusted for Model 3; adjusted for antihypertensive medication, age, BMI, diabetes, dyslipidemia, education level, and income level. NA: not applicable.

Among women current smokers, a significant association was observed between SBP and smoking pack-years [Model 3: B=0.04 (0.01), p<0.001] but not with DBP (p=0.188) and hypertension prevalence (p=0.176) (Table 4). Similar results were observed between cigarettes per day with SBP (p<0.001) and DBP (p=0.217), but showing a significant association with hypertension (p=0.019). SBP and DBP, adjusted for Model 3, showed linear significant correlations with both smoking pack-years (p<0.001) and cigarettes per day (p<0.001) among women current smokers (Figure 2). Women who consumed >30 smoking pack-years showed higher proportion of hypertension (Figure 3). Similar results were observed between adjusted SBP and DBP with cigarettes per day (Figure 3).

For hypertension determination, among current smokers, logistic regressions were performed, and a threshold at 12 cigarettes per day was observed in women (p<0.001) and 11 cigarettes per day in men (p<0.001). Alcohol consumption per day was significantly associated with SBP, DBP and hypertension in all subgroups of current, past, and never smokers (Table 4). For hypertension determination, among current smokers, logistic regressions were performed, and a threshold at 2.71 units per day was observed in women (p<0.001) and 3.19 units per day in men (p<0.001).

Added values in models were observed when including alcohol consumption in models for smoking information, i.e. p<0.001 for smoking pack-years and p<0.001 for cigarettes per day in both genders and for both SBP and DBP (Table 5). However, no added values were observed when including smoking information in models of alcohol consumption (men: SBP, p=0.630 and for DBP, p=0.589; women: for SBP, p=0.619 and for DBP, p=0.921).

**Table 5:** R^2^ values (coefficient of determination) of each multiple linear models for SBP and DBP for men (N=133950) and for women (N=156963)

*Model 3*	*Systolic blood pressure*				*Diastolic blood pressure*			
	*R^2^*	*p*	*p*	*p*	*R^2^*	*p*	*p*	*p*
**Men**								
Smoking pack-years	0.103044	Ref.	0.706	<0.001	0.058722	Ref.	0.841	<0.001
Cigarettes/day	0.104570	0.706	Ref.	<0.001	0.059534	0.841	Ref.	<0.001
Alcohol consumption/day	0.130686	<0.001	<0.001	Ref.	0.084439	<0.001	<0.001	Ref.
Alcohol consumption/day + Smoking pack-years	0.130704	<0.001	-	0.996	0.084674	<0.001	-	0.954
Alcohol consumption/day + Cigarettes/day	0.132628	-	<0.001	0.630	0.086625	-	<0.001	0.589
**Women**								
Smoking pack-years	0.164079	Ref.	0.902	<0.001	0.079354	Ref.	0.905	<0.001
Cigarettes/day	0.163525	0.902	Ref.	<0.001	0.079645	0.905	Ref.	<0.001
Alcohol consumption/day	0.178095	0.002	0.001	Ref.	0.098282	<0.001	<0.001	Ref.
Alcohol consumption/day + Smoking pack-years	0.179101	<0.001	-	0.674	0.098398	<0.001	-	0.962
Alcohol consumption/day + Cigarettes/day	0.179285	-	<0.001	0.619	0.098423	-	<0.001	0.921

* Associations were adjusted for Model 3; adjusted for antihypertensive medication, age, BMI, diabetes, dyslipidemia, education level, and income level.

## DISCUSSION

This study investigated the association between smoking and alcohol consumption with blood pressure according to gender. The findings revealed that SBP and DBP were lower among current smokers than never smokers in both genders and after adjustment for all covariates. Alcohol consumption was significantly and positively associated with higher levels of SBP, DBP and hypertension in men and women. We observed an interaction between smoking and alcohol status in both men and women. We found in current smokers a positive association between smoking pack-years and cigarettes per day with SBP in both genders, but not with DBP, and only for cigarettes per day with hypertension prevalence in both men and women. Synergistic effects were observed by adding alcohol consumption on smoking models in men and women.

### Tobacco smoking and hypertension

Several social factors and individual behaviors can display BP levels among current smokers^18^. However, studies have reported that smoking increases BP^6^. High level of nicotine activates the sympathetic nervous system leading to a release of epinephrine, norepinephrine and vasopressin hormones^19^. Nevertheless, the chronic effect of tobacco smoking remains unclear. Several studies showed that current smokers had lower BP levels compared to non-smokers^3^. However, epidemiological studies showed a dose-dependent effect of smoking on BP^2,6^, even if a meta-analysis highlighted no causal association between BP and smoking heaviness in current smokers^4^. Moreover, former smokers were higher hypertensive than never smokers and the risk of hypertension increased with the number and duration of cigarettes smoked^20^. Nevertheless, there is no consensus regarding the role of chronic tobacco smoking on BP. Tobacco smoking has chemical toxicants which can have detrimental effects and damage^18^. Some findings showed that chronic smokers presented high BP values^21^. More so, since chronic smokers had higher SBP than those hypertensive because of old age^22^. Chronic smoking enhanced several pathways such as oxidative stress, alteration of nitric oxide (NO) and bioavailability, endothelial dysfunction, and then increased BP^7,20^. The effect of a chronic tobacco smoking on BP can be explained by the damage caused by nicotine and carbon monoxide, two main compounds of tobacco^23^. Nicotine leads to a vasoconstriction and vasoparalytic effects. In parallel, carbon monoxide affects the arterial wall and leads to irreversible damage on arteries leading to increased BP. Former smokers presented a decrease in BP only if carbon monoxide did not already affect the arterial wall^24^. Chronic carbon monoxide exposure, as in chronic smokers, presented irreversible alterations of blood vessels^25^. However, to date, few reports have documented and shown an association between smoking and onset of hypertension. This relationship should be mainly documented with clear evidence^4^.

### Alcohol and hypertension

Consistent with previous studies, our findings highlight that alcohol consumption is significantly associated with increased BP^26^. However, the association between hypertension and alcohol consumption remains unclear in women^27^. A meta-analysis study assessed the presence of a gender-specific relationship between alcohol consumption and hypertension^9^. In our study, we found different thresholds for alcohol consumption and hypertension determination: 3.19 units/day for men, and 2.71 units/day for women. These findings are consistent with a recent meta-analysis showing that alcohol consumption increased the risk of hypertension in men for consumption of more than 1 to 2 drinks/day (when considering one red wine drink=2 units) while heavy consumptions were significant in both genders^28^. Thus, a gender dose-response relationship was observed between alcohol consumption and hypertension. One of the possible explanations could be the many drinking occasions with an average of alcohol consumption among men than women (Table 1). The frequency of alcohol consumption presented different effects on BP^29^. Previous studies have shown that the consumption amount of alcohol was associated with high BP and that the reduction in alcohol intake lowered BP in a dose-dependent response^6^. Alcohol consumption may be responsible for vasoconstriction of blood vessels, increased heart rate, activation of the sympathetic nervous system and loss in magnesium^6^.

### Synergistic effects of tobacco smoking and alcohol consumption on hypertension

Drinking and smoking behaviors generally occur together^30^. Alcohol consumption can affect the relationship between smoking and BP, whereas the relationship between alcohol consumption and BP did vary by smoking status^31^. Thus, the synergistic effects remained unclear. The fact that both alcohol consumption and tobacco smoking can interact with the sympathetic nervous system could explain a synergistic effect. However, only one study has shown that the combine reduction in alcohol consumption and tobacco smoking was associated with reduction in hypertension^32^. Moreover, very few studies have focused on this possible interaction to highlight this possible synergistic effect among current smokers^6,31^. Our study showed an added effect of alcohol consumption on smoking pack-years and cigarettes per day in current smokers. The alcohol consumption effect could be reinforced by the neurochemical action of nicotine, explaining the added value of alcohol in smokers^33^. However, we found no added effect of tobacco consumption on alcohol consumption. Alcohol is known to be highly associated with abdominal obesity and thus increased risk of obesity^34^ whereas tobacco smoking was correlated with lower BMI^35^. As BMI, after age, was one of the main factors of increased risk of hypertension^36^, the absence of added effects of tobacco use on alcohol consumption observed could be explained by these inverse interactions with BMI.

### Strengths and limitations

The main strength of this study is the very large sample size of the cohort. The cross-sectional observational design limits the relationship of causality. Reverse causation cannot be ruled out. The UK Biobank study showed a low response rate of 5.5% and possible volunteers bias may be involved. Nevertheless, given the large sample size and high internal validity, these are unlikely to affect the reported associations. In addition, the study cohort consisted of middle-aged European participants, so our findings may not be generalized to other age groups and ethnic populations. In addition, the UK Biobank used standardized protocols to collect anthropometric data including BP measurements; this ensures replication of data collection for all volunteers regardless of when, where and by whom they are performed and adds validity to our results. However, our study presents some limitations. Socioeconomic data were collected by self-reporting. Medical history and comorbidities have been collected by self-reporting and physician verification during medical examination in health centers. The cross-sectional design of the study may represent a limitation since reverse causation cannot be excluded. Smoking pack-years, cigarettes per day and alcohol consumption were self-reported by questionnaire. Moreover, periods of quitting smoking have not been included in calculating smoking pack-years, due to a lack of information about the duration period of stop smoking. Due to the adjustment for several factors which are causal pathways, a collider bias should be considered for the interpretation of the results observed.

## CONCLUSIONS

Our findings showed lower BP in current smokers than never smokers in both genders. Nevertheless, among current smokers smoking pack-years, cigarettes per day and alcohol consumption were associated with higher BP. Synergistic effects of alcohol consumption on tobacco smoking were observed for SBP and DBP. Although the relationships remained modest, these risk factors could be considering to be part of the public health policies to reduce hypertension risk.

## Data Availability

The data supporting this research cannot be made available for privacy or other reasons. UK Biobank data are available through the UK Biobank Access Management System (UK Biobank Access Management System: http://www.ukbiobank.ac.uk/register-apply/).
